# Reevaluating the role of beta2-microglobulin: new insights on selective vulnerability in ALS pathology

**DOI:** 10.1007/s00401-026-03024-3

**Published:** 2026-05-29

**Authors:** Melanie Leboeuf, Jik Nijssen, Laura Helen Comley, Julio Cesar Aguila Benitez, Irene Mei, Silvia Gómez Alcalde, Ramón A. Muñoz de Bustillo-Alfaro, Vlad Radoi, Susanne Nichterwitz, Christoph Schweingruber, Abraham Acevedo Arozena, Eva Hedlund, Staffan Cullheim

**Affiliations:** 1 Department of Biochemistry and Biophysics, Stockholm University, Svante Arrhenius v. 16C, 106 91 Stockholm, Sweden; 2 Department of Cell and Molecular Biology, Karolinska Institutet, Biomedicum, 171 77 Stockholm, Sweden; 3 Department of Neuroscience, Karolinska Institutet, Biomedicum, 171 77 Stockholm, Sweden; 4 Hospital Universitario de Canarias, Instituto de Investigación Sanitaria de Canarias, ITB-ULL, Tenerife, Spain

**Keywords:** Motor neuron, Amyotrophic lateral sclerosis, Beta2-microglobulin, MHC-I, Selective vulnerability

## Abstract

Amyotrophic lateral sclerosis (ALS) is characterized by the selective loss of motor neurons (MNs). Why these neurons are particularly vulnerable in ALS remains unclear, as does why certain MN groups remain resistant throughout the disease course. We investigated the role of the human leukocyte antigens (HLAs) and beta2-microglobulin (β2m) in MN susceptibility to ALS, given their reported involvement in both prolonging and shortening disease progression. Loss of HLAs in ALS has also been shown to increase MNs vulnerability to toxicity exerted by activated astrocytes. RNA sequencing of control tissues demonstrated that disease-resistant oculomotor neurons (OMNs) and Onuf’s MNs exhibited *β2m* and *HLA* mRNA levels comparable to those of vulnerable spinal MNs, suggesting that baseline differences in these transcripts do not explain the differential vulnerabilities of these MN groups. However, HLA protein levels showed an inverse correlation with spinal MN size, with the large MNs, those lost early in ALS, displaying the lowest HLA expression. HLA protein levels were also reduced in spinal MNs from end-stage ALS patient tissues, while remaining relatively unchanged in OMNs. In contrast, spinal MNs uniquely exhibited significant upregulation of *β2m* and *HLA-C* transcripts during disease, likely reflecting a protective compensatory response. Together, these findings suggest that β2m and HLAs may contribute to spinal MN vulnerability in ALS. To assess their functional role, β2m knockout mice were crossbred with SOD1G93A ALS mice. Loss of β2m did not alter life span of the ALS mice, but led to partial preservation of lumbrical muscle innervation that was insufficient to maintain motor function. Analysis of GFAP immunoreactivity revealed marked neuroinflammation activation in the spinal cords of β2m knockout mice. As these mice retain normal MN numbers and life-span, this indicates that loss of functional MHC-I, even in the presence of astrocyte activation, is insufficient to cause MN disease. Furthermore, β2m knockout significantly increased GFAP activation in SOD1G93A mice, but did not further exacerbate disease progression, suggesting that loss of functional MHC-I does not necessarily render MNs more vulnerable to astrocyte toxicity. Overall, these findings indicate that β2m and HLAs are dynamically regulated in ALS, and may influence MN vulnerability, but they are not major disease modifiers in ALS.

**Supplementary Information:**

The online version contains supplementary material available at 10.1007/s00401-026-03024-3.

## Introduction

**Fig. 1:**
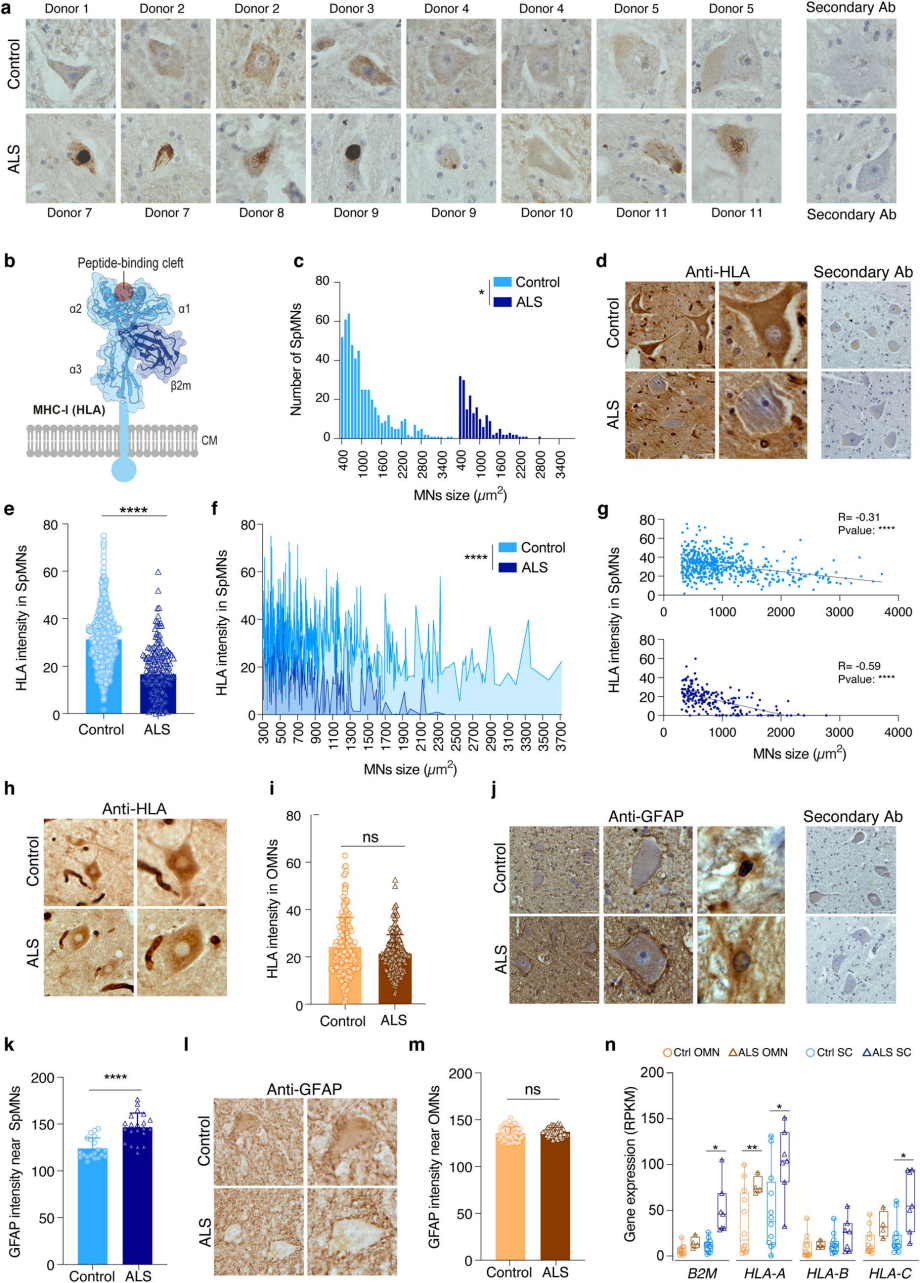
Motor neuron loss in ALS is correlated with a decrease in HLA protein, increased GFAP immunoreactivity and upregulation of *B2M* and HLA-A/C mRNAs. **a** Representative images of immunohistochemical staining for pTDP-43 (phosphorylated TDP-43) in spinal cord tissue from control and ALS post-mortem donors, revealing pTDP-43 aggregates in the majority of ALS cases. **b** Schematic of the MHC-I molecule. **c** Measurement of motor neuron (MN) area (μm^2^) in control and ALS spinal cord donor tissues shows a loss of MNs across sizes (*P* < 0.05, Kolmogorov-Smirnov test). **d** Representative images of immunohistochemical staining against HLA in human control and ALS spinal cord *post mortem* donor tissues. **e** Quantification of HLA immunoreactivity from antibody-staining (in **d**) shows a significant decrease in ALS end-stage patient spinal MNs (SpMNs) compared to control (*P* < 0.0001, Mann–Whitney Unpaired t-test, data are expressed as the mean ± SD). **f** HLA intensity in SpMNs correlated with cell size (*P* < 0.0001, Kolmogorov-Smirnov Unpaired t-test). **g** HLA intensity and SpMN size are inversely correlated both in human control and ALS spinal cord *post mortem* donor tissues (Ctrl: R = −0.31, *P* < 0.0001, Spearman correlation, ALS: R = −0.59, *P* < 0.0001, Spearman correlation). **h** Representative images of immunohistochemical staining against HLA in oculomotor neurons (OMNs) in human control and ALS brain donor tissues. **i** Quantification of HLA immunoreactivity from antibody-staining (in **h**) shows no difference in HLA intensity in OMNs between control and ALS donors (*P* = 0.06, Mann–Whitney, data are expressed as the mean ± SD). **j** Representative immunohistochemical staining against GFAP in human control and ALS spinal cord donor tissues. **k** Quantification of GFAP immunoreactivity in the spinal cord shows an increase in ALS patients (*P* < 0.0001, Mann–Whitney, data are expressed as the mean ± SD). **l** Representative images of immunohistochemical staining against GFAP in human control and ALS donor tissues in the OMN region of the midbrain. **m** Quantification of GFAP immunoreactivity shows no difference between control and ALS donors in the OMN region of the midbrain (*P* = 0.11, Mann–Whitney, data are expressed as the mean ± SD). **n** RNA sequencing of MNs shows an increase in *β2M* levels in SpMNs in ALS, and an increase in *HLA-A* in OMNs and spinal MNs with disease as well as an increase in *HLA-C* in SpMNs in ALS. (N = min. 4 samples per group, each representing one individual. P values: *< 0.05, **< 0.01, obtained with Welch test)

Major histocompatibility complex class I (MHC-I) molecules are present as transmembrane glycoproteins on the surface of all nucleated cells. MHC-I proteins display peptide fragments to circulating cytotoxic T cells and natural killer cells (NKs). This antigen presentation is essential for immune defense against intracellular pathogens and cancer [1]. In case the peptide fragments derive from non-self-proteins, this may trigger an immediate detrimental response from the immune system. However, alterations in MHC-I expression can have unintended consequences; downregulation can trigger NK cell-mediated cytotoxicity [2], whereas aberrant upregulation is implicated in autoimmunity and neurodegeneration [3]. Structurally, MHC-I consists of an alpha heavy chain, which has two domains to which peptides bind, an immunoglobulin (Ig)-like domain and a transmembrane region with a cytoplasmic terminus. The heavy chain of class I molecules is encoded by genes at the HLA-A, HLA-B or HLA-C loci in humans, and is linked to a conserved light chain, beta-2-microglobulin (β2m), which is required for MHC-I stability and surface expression [4] (Fig. 1b). Beyond its classical immunological role, MHC-I has been increasingly recognized as a key player in the nervous system, where it contributes to synaptic pruning, neurodevelopment, plasticity [5–10] and may be involved in neurological disorders [11–13]. Motor neurons (MNs), in particular, exhibit high expression of mRNAs for MHC-I, including β2m, under physiological conditions [14], with further upregulation following axonal injury where MHC-I protein accumulates at the end of cut axons [14, 15]. The mouse gene H2-Db also increases in MN somas after axotomy (both at mRNA and protein level) and in axons [15]. These findings suggest that MHC-I may influence neuronal survival, degeneration and regeneration. The presence of high levels of MHC-I and β2m in MNs has spurred an interest in finding a role for these proteins in degenerative MN diseases, in particular amyotrophic lateral sclerosis (ALS). While MHC-I and β2m are suspected to play a role in ALS, research has yielded diverse findings regarding their expression and function. In an ALS mouse model, over-expressing a disease-associated variant of superoxide dismutase 1 (SOD1G93A) resulted in increased *β2m* mRNA levels in spinal MNs of symptomatic mice. When the SOD1G93A mice were crossed with β2m knockout mice, survival was decreased due to a shorter disease duration, indicating that β2m was protective in ALS and that upregulation of β2m in MNs with disease may be a protective compensatory response. However, this study did not investigate the level of MHC-I or β2m on the protein level [16]. Another study reported that HLAs were reduced in MNs in symptomatic SOD1G93A mice and that MHC-I levels were completely abolished in MNs in end-stage ALS patient tissue [17]. It was moreover demonstrated that astrocytes reduced HLA expression on MNs, rendering these neurons more susceptible to astrocyte-induced cell death. Overexpression of HLA-C (H2-K1 in mouse) in MNs, but not HLA-A or HLA-B, could rescue this susceptibility [17]. The abovementioned studies suggest that the presence of β2m or HLAs, and presumably then MHC-I, on MNs may have a beneficial effect on MN survival in ALS. On the other hand, a third study suggested that while the activation of MHC-I in the peripheral nervous system of SOD1G93A mice preserves muscle innervation and motor function, the effects in the central nervous system (CNS), mediated by the interaction of microglia expressing MHC-I with CD8^+^ T cells accelerates MN death and reduce the overall survival of SOD1G93A mice and thus removing β2m increased survival of the mice [18]. These disparities highlight the complexity of MHC-I regulation and possible function in ALS and underscore the need for further investigation.

To address this, we analysed MHC-I expression in ALS across human and mouse datasets. Our analysis shows that there is an inverse correlation with HLA protein levels and MN size in the spinal cord. We also show that HLA protein levels are reduced in spinal MNs with ALS and that this is unique to vulnerable MNs while OMNs as a group maintain their expression throughout disease. We also demonstrate a compensatory upregulation of *β2m* and *HLA-C* mRNAs only in ALS-vulnerable spinal MN, which may be part of an insufficient neuroprotective response. To directly assess the impact of β2m on ALS disease progression, we generated SOD1G93A;β2m−/− mice and evaluated their motor function, disease course, neuronal survival and synapses with muscle. Our findings demonstrate that loss of β2m had no impact on motor function in either males or females, and did not affect survival of SOD1G93A mice. Nonetheless, while neuromuscular junction (NMJ) innervation remained unchanged in tibialis anterior and soleus muscles, the lumbrical muscles of SOD1G93A;β2m−/− mice exhibited increased NMJ stability, suggesting a distal protective effect, that was not reflected at the phenotypic level.

## Materials and methods

### Bioinformatics analysis of RNA sequencing and microarray data

Processed files of microarray datasets were obtained from GEO, including Brockington et al. 2013 (GSE40438, [19])*,* Kaplan et al. 2013 (GSE52118, [20]), and Shadrach et al. 2021 (GSE162028, [23]), together with processed gene count or intensity tables from Perrin et al. [21], Lobsiger et al. [22], Allodi et al. [24], Nizzardo et al. [25], retrieved from the original authors. To minimize technical and biological heterogeneity, datasets were analysed independently according to species, disease stage, and experimental platform (RNA sequencing vs microarray). Direct merging of dataset was avoided. For RNA sequencing datasets, Bandyopadhyay et al. [26] and Mei et al. [27], were mapped to mm39 genome assembly using STAR (version 2.7.0e) [28], and gene level expression was quantified using rpkmforgenes.py with Ensembl gene annotation (mm39). Expression values were normalized prior to downstream analysis. Differential expression analyses were performed within each dataset separately, comparing biologically matched conditions (e.g., ALS versus control within the same species and stage). Where applicable, normalisation procedures and statistical models account for differences in sequencing depth and library composition.

### Ethics statement for the use of human tissues

The use of human post mortem tissues was approved by the ethical review board of Sweden (Etikprövningsmyndigheten) and was conducted in compliance with the code of ethics of the World Medical Association (Declaration of Helsinki). Human samples were retrieved from the Netherland’s Brain Bank (NBB, http://www.brainbank.nl), who in turn retrieved samples with the written informed consent from the donors or next of kin (Table 1). Cases classified as controls from the NBB did not present with dementia or any other neurological disease, while ALS cases had a diagnosis of definite ALS.

### Ethics statement regarding animals and animal models used

All animal procedures were approved by the Swedish ethical council (Stockholms Norra Djurförsöksetiska nämnd) in compliance with the code of ethics of the World Medical Association (Declaration of Helsinki) and national legislation and institutional guidelines. Mice were housed according to standard conditions, with access to food and water ad libitum and a dark/light cycle of 12 h at the laboratory animal facility, Karolinska Institutet. To investigate the role of β2m in ALS we used the strain B6.129P2-β2m tm1/Unc/J, Jackson Laboratory, Stock Number 002087, congenic on a C57BL/6J background. These mice are homozygous for the *B2m*^*tm1Unc*^ targeted mutation (β2m-KO) and fail to express MHC class I protein on the cell surface and are grossly deficient in CD45− CD8+ T cells [29]. To generate experimental ALS-mice with different doses of β2m we crossed β2m−/− females with transgenic males of the SOD1G93A colony (B6.Cg‐Tg(SOD1*G93A)1Gur/J; Jackson Laboratory Stock, Number 004435). These mice overexpress mutant human SOD1 protein leading to an ALS‐like phenotype [30]. Litters of this first cross (F1), always heterozygous for β2m (β2m±), were crossed to generate all possible genotype combinations with the mutant SOD1 background (F2): SOD1G93A;β2m+/+, SOD1G93A;β2m± and SOD1G93A;β2m−/−. Some experimental animals, normally produced at lower frequency, were also generated by crossing: β2m−/− females from the original colony with mutant SOD1-transgenic males from the F1 (SOD1G93A;β2m+/−) or β2m+/+ females with males (SOD1G93A;β2m+/+), both from the F2.

Genotyping of both colonies and experimental animals was carried out by regular PCR following The Jackson Laboratory protocols while qPCR was routinely used to follow transgenic SOD1 copy number of male breeders (also following Jackson’s recommendations).

### Behavioural analysis and disease staging

Body weight of experimental animals was measured twice a week from postnatal day (P) 56. From week 8 (~ P56) to week 20 (~ P140), animals were scored weekly for the extension reflex of their hind limbs and challenged with the inverted grid test. In the extension reflex test, the mice were suspended from the tail and their hind limb extension reflex was scored from 3.0 (normal) to 1.0 (ALS condition) with 0.5 interval points during disease progression. In the inverted grid test, the time a mouse holds onto a grid was measured. Prior to any measurements, mice were first habituated to the grid by being placed onto it on two separate days, performing the procedure, without taking any actual measurements. Then for the experimental days, mice were placed on the inverted grid once without taking any measurement and subsequently with measurements. The maximum duration of the assay was 120 seconds (s) (healthy, normal situation after task learning). If the animal did not reach the 120 s, another trial was carried out after 1–2 min of recovery. The longest time measured was selected as the behavioural performance for that week. Some records were excluded from the inverted grid analysis since a few animals learn to jump after just a few seconds of trial. Ultimately, maximum performance of each mouse was set at 100% and decreased performances normalised against this value. For assessment of survival, animals were kept until end-stage. Once the animals got severely impaired hind limbs (typically around ~ P140), they were monitored twice a day and scored using the KI assessment checklist to follow animal health status. From this point water bottles with long drinking spouts were provided to facilitate water access. In addition, animals received soft gel food (Scanbur) on the cage floor. End stage was defined as the inability of the animals to right themselves within 15 s after being placed on either side.

### Tissue collection, processing and analysis

For muscle analysis, mice were sacrificed by inhalation of CO_2_. Lumbrical muscles (from the plantar surface of the hind-paw), soleus and tibialis anterior (TA) were dissected in 0.1 M phosphate buffered saline (PBS) and fixed in 4% paraformaldehyde (PFA) (Sigma-Aldrich) for 30 min for NMJ analysis. Lumbricals were analysed in whole-mount, whereas soleus and TA muscles were sectioned at 30 μm thickness. For CNS immunohistochemistry, animals were anesthetised with avertin (2,2,2-Tribromoethanol; Sigma-Aldrich) and perfused intracardially with PBS followed by 4% PFA. Brains and spinal cords were dissected and postfixed (for 3 and 1 h, respectively), cryoprotected in sucrose and sectioned (30 μm). Tissues were imaged on a Zeiss LSM700 or 800 confocal microscopes and a Zeiss Axio imager M1 microscope.

### Immunofluorescent analysis of astrocytes and CD8+ T cells in mouse spinal cord tissue

Spinal cords were cut at the cervical level, mounted in OCT and sectioned at 30 µm-thickness in a cryostat (2–3 animals per genotype and sex). Sections were attached to Superfrost Plus slides (ThermoFisher Scientific) and stored at -20ºC. Blocking of mouse spinal cord sections was performed in 5% donkey serum in 0.3% triton X-100 (Sigma Aldrich) in PBS. The tissue was subsequently incubated with primary antibodies in 5% donkey serum in 0.3% triton X-100 in PBS overnight at 4 °C. The primary antibodies used were mouse anti-GFAP (Sigma, G3893, 1:500), and rat anti-CD8 (AbD Serotec, MCA341R, 1:50). Sections were subsequently washed with PBS and incubated with secondary antibodies Alexa-488 donkey anti-rabbit, Alexa-568 donkey anti-mouse (Invitrogen, 1:2000) and Hoechst (ThermoFisher Scientific, H3570, 1:2500) in 5% donkey serum in 0.3% triton X-100 in PBS for 1 h at room temperature. Next, slides were washed with PBS and mounted using Fluoromount G mounting media (ThermoFisher Scientific, 00-4958-02). Images of spinal cord sections were acquired with an inverted confocal (LSM800-Airy) microscope (Zeiss) using a 20× objective. Images were captured in the ventral horn of the spinal cord where spinal MNs are located. For GFAP quantification, at least three images per sex and genotype were taken and the mean intensity of each image was measured using the ImageJ software.

### NMJ innervation in tibialis anterior, lumbricals and soleus muscles

Muscle was permeabilised with 4% triton X-100 for 1 h. Blocking was performed with 10% donkey serum in 0.1% triton X-100 (Sigma Aldrich) in PBS and the tissue was subsequently incubated with primary antibodies in 10% donkey serum and 0.1% triton X-100 in PBS for 48 h. Primary antibodies used were mouse anti-synaptic vesicle protein (DSHB, SV2, 1:100) and mouse anti-neurofilament 165 kDa (DSHB, 2H3; 1:50). Next, slides were washed with PBS and incubated with secondary Alexa-488 donkey anti-mouse antibodies in PBS (1:500; Invitrogen) for 3 h at room temperature. Finally, to visualise endplates, α-bungarotoxin (α-BTX) staining was performed for 15 min using tetramethyl-rhodamine isocyanate-conjugated α-BTX (1:1,000; Invitrogen). Slides were coverslipped using Mowiol 488 mounting media (Sigma-Aldrich). Innervation of the NMJ was determined by analysing a minimum of 50 endplates across the muscle. Each endplate within a field of view was categorised as either fully occupied (the presynaptic terminal completely overlies the endplate), partially occupied (the presynaptic terminal partially covers the endplate) or vacant (no presynaptic staining overlies the endplate). All analyses and quantifications were performed blind to the genetic status of the muscles.

### Quantification of GAP-43 expression at the NMJ

For analysis of GAP-43 expression at the NMJ the first and second deep lumbrical muscles from the hind paw of P140 SOD1G93A;β2m+/+ and SOD1G93A;β2m−/− mice were dissected whole mount to preserve the entire innervation pattern. After fixation, tissue was permeabilised in 4% triton-X 100 (Sigma; 0.1%) in 0.1 M PBS for one hour and blocked in 10% donkey serum (Jackson Immuno Research) and 0.1% triton-X 100 in 0.1 M PBS for a further hour at room temperature. Muscles were incubated over two nights at 4 °C in blocking solution with primary antibodies directed against neurofilament (DSHB, 2H3, 1:50) and GAP-43 (Millipore, AB5520 1:250) in order to visualize axons and regenerating neurons, respectively. Muscles were then washed twice for 30 min in 0.1% triton-X 100 and 10% donkey serum in 0.1 M PBS and incubated for 2 h with Alexa Fluor 488 donkey anti-mouse and Alexa Fluor 568 donkey anti-rabbit secondary antibodies in 0.1 M PBS (1:500, Life Technologies). Muscles were washed in 0.1 M PBS for 30 min and then exposed to Alexa Fluor 647 α-BTX (1:1000, Life Technologies) for ten minutes to label post-synaptic acetylcholine receptors. Muscles were then whole-mounted in Mowiol 488 (Sigma-Aldrich) on glass slides and cover-slipped for subsequent imaging. Imaging was performed on laser scanning confocal microscopes (Zeiss LSM700 and LSM800). At least 50 NMJs were individually categorized per muscle per mouse based on the level of GAP-43 expression at each one (minimum of 65 NMJs per muscle). GAP-43 levels were classed as distinct (bright and defined staining overlying the endplate), diffuse (faint and undefined staining, or only partially overlying the endplate) or devoid (no GAP-43 overlying the endplate), as previously described [31]. All analyses and quantifications were performed blind to the genetic status of the muscles.

### Motor neuron counts in mouse spinal cords

For quantification of spinal MN somas, end-stage SOD1G93A;β2m+/+, SOD1G93A;β2m+/−, SOD1G93A;β2m−/− and age-matched WT;β2m−/− mice (2–4 animals per genotype and sex) were analysed. Spinal cords were cut at the lumbar level, mounted in OCT and sectioned (30 µm-thickness) in a cryostat. Lumbar sections were attached to Superfrost Plus slides (Thermo Scientific) and stored at −20 °C. Prior to staining, slides were air-dried, fixed in 25% ethanol for 2 min and Nissl-stained using 2% Cresyl Violet acetate (Sigma-Aldrich, in 25% ethanol, pH8) for 2 min. Slides were washed in water for 1 min and then dehydrated in ethanol 25, 50, 70, 95 and 100% for 2 min each. Finally, slides were moved to Xylene for 3 min, quickly air-dried, mounted using Mountex (Histolab) and kept at room temperature. MNs were counted in a minimum of six pairs of ventral horns at the lumbar level of the spinal cord. Cells were counted on a Zeiss Axio Imager M1 Upright microscope using the Q-capture software. In order to selectively count alpha-MNs, only cells located in the ventral horn with a cell body diameter greater than 20 μm and a clear nucleolus were included. To ensure accurate assessment of soma size the projected image in Q-capture was calibrated to match a grid which could be superimposed over the image of the ventral horn so that each box measured precisely 20 μm. This way only cells with a cell body diameter greater than 20 μm were counted.

### Immunohistochemistry in human tissues

Paraffin-embedded human sections of 12 μm were incubated at 60 °C for 30 min, then moved to xylene for 10 min at room temperature. They were sequentially rehydrated with 100% ethanol (2 × 10 min), 95% ethanol (2 × 10 min), 70% ethanol (5 min), 50% ethanol (2 min), 25% ethanol (2 min), and finally rinsed with deionised water for 1 min. Slides were heated in citrate buffer (10 mM Sodium Citrate, Sigma-Aldrich S4641; 0.05% Tween 20, Sigma-Aldrich P7949; pH 6.0) at 95 °C for 20 min and washed in PBS for 30 min. Endogenous peroxidases were quenched using a 50% methanol, 3% hydrogen peroxide solution (H_2_0_2_; Sigma Aldrich, 216763) in PBS for 10 min, followed by a 5-min PBS wash. Sections were blocked with 10% serum in PBS with 0.1% Triton X-100 (Sigma Aldrich) for 1 h, and then incubated with primary antibody in PBS with 0.1% Triton X-100 and 10% serum for 72 h at 4 °C. Primary antibodies used for immunohistochemistry in human tissue were rabbit anti-GFAP antibody (Dako, Z0334, 1:200), mouse anti-HLA Class 1 ABC antibody (Clone EMR8-5, Abcam, ab70328, 1:100), mouse anti-pSer409/410 TDP-43 (Clone 11-9 CosmoBio, CAC-TIP-PTD-M01A, 1:5000) and mouse anti-CD68 (Abcam, ab955, 1:100). For HLA staining, both blocking and primary antibody incubation were carried out without Triton X-100. As negative controls for the antibody stainings we also included tissue sections that did not receive any primary antibodies, but only secondary antibodies. After washing three times in PBS, slides were incubated with Biotin-SP affinitypure donkey anti-rabbit IgG (Jackson ImmunoResearch, 711.065.152, 1:100) or anti-mouse IgG secondary antibody (Jackson ImmunoResearch, 715.065.151, 1:100) in PBS overnight at 4 °C. The avidin–biotin complex (ABC) (VECTASTAIN Elite ABC-HRP Kit, Vector Laboratories PK-6100) solution was applied for 1 h at room temperature, followed by washes in PBS (3 × 10 min). DAB staining was performed using the Vector DAB substrate kit (Vector Laboratories, SK-4100), with a final wash in ddH₂O for 10 min. Nuclei were counterstained with Myers hematoxylin (Sigma Aldrich, GHS132) for 2 min, rinsed in ddH₂O, then treated with 70% ethanol containing 36 mM HCl to remove background staining, followed by another ddH₂O rinse. Dehydration was completed with sequential ethanol treatments for 2 min each (25, 70, 75, 95, and 100% ethanol), xylene (2 × 2 min), and mounted using DPX new non-aqueous mounting medium (Sigma Aldrich, 1005790500)*.* Images of spinal cord and brain sections were acquired with a Zeiss Axio Observer 7 microscope under bright‐field conditions using a 20× objective.

### Protein level analysis in human tissue

The human *post mortem* cases and tissues used for quantification of anti-HLA and anti-GFAP stainings are listed in Table 1. For quantification of HLA staining intensity in MNs in spinal cord and midbrain the following procedures were followed. Specificity of the staining was evaluated by performing secondary antibody–only controls in all donors, using mouse secondary antibodies for HLA staining and rabbit secondary antibodies for GFAP staining. No specific staining was observed under these conditions in any tissue. This was determined by assessing the overall staining intensity across entire sections and comparing it to sections from the same patient in which primary antibodies were included. Neurons located in the ventral horn of the spinal cord were manually outlined using the ImageJ software (NIH, Bethesda, MD), and the intensity of the staining was measured in somas with an area of 300 μm^2^ or greater, which were considered to be MNs based on size and location, and the background staining was subsequently subtracted. OMNs were identified based on their anatomical location in midbrain tissue sections and morphological features, manually outlined in ImageJ, and staining intensity in somas was quantified after background subtraction. The absolute majority of OMN somas had an area ranging from 200 to 1200 μm^2^ (Supplementary Fig. 1e). The number of donor samples used and MNs quantified for HLA staining are outlined in Supplementary Table S1, as well as the sections used for anti-GFAP and anti-CD-68 staining in Supplementary Table S2.

**Table 1:** Information on human *post mortem* tissue samples used

Subject	Age	Dagnosis^a^	Sex	PMI (h)	Age of clinical onset (years)	Disease duration (months)	Site of onset	Tissue used in study	ALS neuropathology from Netherland’s Brain Bank	pTDP43 pathology
Donor 1	71	Control	M	7:40	N/A	N/A	N/A	Spinal cord, midbrain	N/A	No
Donor 2	90	Control	F	5:25	N/A	N/A	N/A	Spinal cord	N/A	No
Donor 3	62	Control	M	8:00	N/A	N/A	N/A	Spinal cord	N/A	No
Donor 4	95	Control	F	5:40	N/A	N/A	N/A	Spinal cord, midbrain	N/A	No
Donor 5	71	Control	F	7:10	N/A	N/A	N/A	Spinal cord, midbrain	N/A	No
Donor 6	99	Control	F	4:15	N/A	N/A	N/A	Spinal cord	N/A	N.I
Donor 13	87	Control	F	5:00	N/A	N/A	N/A	midbrain	N/A	N.I
Donor 14	88	Control	M	7:00	N/A	N/A	N/A	midbrain	N/A	N.I
Donor 7	62	ALS	M	6:55	61	20	Bulbar	Spinal cord	Betz cells show signs of degeneration, clear loss of CNXII MNs, spinal MNs, TDP-43 pathology	Yes
Donor 8	70	ALS	M	5:25	67	45	Bulbar	Spinal cord, midbrain	Loss of MNs in CNXII and spinal cord	Yes
Donor 9	49	ALS	F	3:45	47	18	Bulbar & limbs	Spinal cord	Severe loss of CNXII MNs, subtle loss of spinal MNs	Yes
Donor 10	67	ALS	M	6:35	57	123	Limbs	Spinal cord	Substantial loss of MNs in spinal cord and CNXII, no TDP-43 staining, microgliosis	No
Donor 11	71	ALS	M	6:45	70	19	Bulbar	Spinal cord	Slight loss of MNs in CNXII. TDP-43 pathology. Loss of spinal MNs	Yes
Donor 15	33	ALS	M	5:55	31	27	Bulbar and limbs	midbrain	Loss of MNs in CNXII and spinal cord	N.I
Donor 16	68	ALS	F	5:00	66	18	Bulbar	midbrain	Loss of spinal MNs, gliosis, ubiquitin inclusions, CD68+ cells in spinal cord	N.I

*N/A* non-applicable, *N.I* not investigated

^a^All controls were classified as “non-demented controls” by the NBB

For anti-GFAP and anti-CD-68 stainings around spinal MNs, images were taken in the ventral horn of the spinal cord where MNs could be identified, and the mean intensity of each image was measured using the ImageJ software. For GFAP quantification around OMNs, images were taken in midbrain sections, and staining intensity for each image was calculated as the average of the mean intensity from three equal-sized regions of interest. The number of donor tissue samples, and sections used from these as well as images captured and quantified are listed in Supplementary Table S2.

### Statistics

Data was collected and analysed using GraphPad Prism software. Statistical significance was determined as follows. For the MNs size analyses the Kolmogorov–Smirnov test was performed. For the measurement of staining intensities in MNs the Mann–Whitney was performed. For correlations of intensity with MN size, Spearman correlation was performed. For the mRNA levels and NMJ analyses, experimental data were compared by 2-way ANOVA. For behavioural analysis, mice were compared using ANOVA (Kruskal–Wallis test) followed by Dunn–Bonferroni post hoc correction. The survival comparison was performed using Mantel-Cox test. For analyses of MN numbers, one‐way ANOVA followed by a post hoc Tukey was performed.

## Results

### HLA protein is selectively reduced in vulnerable MNs in ALS patients but is not sufficient to induce MN loss

In light of the report that HLAs are reduced in spinal MNs in symptomatic SOD1G93A-ALS mice and that MHC-I levels were abolished in spinal MNs in end-stage ALS patient tissue [17], we wanted to investigate if HLAs (Fig. 1b) could play a role in selective MN vulnerability. Towards this goal, we analysed the number of MNs in *post mortem* tissue from control and ALS donors together with their HLA levels via immunohistochemistry. Control tissues originated from patients that were never diagnosed with a neurological disease, including dementia and ALS (Table 1). Analysis of anti-pSer409/410 TDP-43 (pTDP-43) pathology in spinal cord tissues demonstrated the presence of both round and skein-like inclusions in the cytoplasm in four out of five ALS cases examined, while control patient material showed no pTDP-43 pathology (Fig. 1a). Notably, donor 10 (ALS), that lacked pTDP-43 pathology, had a substantially longer disease duration than all other cases (Table 1). We observed a loss of MNs across all sizes in ALS patients, with larger diameter MN somas (> 2800 μm^2^) being completely abolished (Fig. 1c, *P* < 0.05). To understand the role of MHC-I in ALS, we analysed HLA protein levels in spinal MNs using immunohistochemistry. Quantification demonstrated a significant reduction in HLA protein in the remaining spinal MNs of ALS patients compared to controls (Fig. 1d–e, *P* < 0.0001) and Supplemental Fig. 1a–c) which was unrelated to the donor age (Supplemental Fig. 1d, Spearman correlation between increasing age and HLA levels were *R* = 0.2, *P* = 0.78 for control tissues and *R* = 0.3,* P* = 0.68 for ALS tissues). The decline in HLA levels in ALS was apparent across MN sizes (Fig. 1f, *P* < 0.0001). Furthermore, there was a clear inverse correlation between HLA protein expression and MN soma size in control and ALS tissues, with the largest most vulnerable MNs showing the lowest HLA level (Fig. 1g, Ctrl (top panel): Spearman correlation, *R* = −0.31, *P* < 0.0001; ALS (bottom panel): Spearman correlation, *R* = −0.59, *P* < 0.0001). Donor 10 did not differ from the other ALS cases in terms of loss of HLA protein levels in MNs (Supplementary Fig. 1a, c). Thus, there was no evident correlation between pTDP-43 pathology and loss of HLA within spinal MNs.

We next investigated HLA expression in OMNs, which are resistant to degeneration in ALS. Quantification of the number of OMNs, demonstrated, as anticipated, no difference between control and ALS cases (Supplemental Fig. 1e). HLA protein levels appeared overall maintained in OMNs at end-stage of disease (Fig. 1h, i, Supplemental Fig. 1f). Nonetheless, when analysing HLA expression as a function of MN size, it became evident that larger OMNs expressed the highest levels of HLA in healthy tissue, while in ALS, the largest OMNs showed the lowest HLA expression (Supplemental Fig. 1g). This indicates that while the overall average expression is maintained in ALS (Fig. 1i), the largest OMNs appear to downregulate HLA protein levels with disease.

Subsequently, we evaluated GFAP levels, as a proxy for activated astrocytes, as it was demonstrated that loss of HLA rendered MNs susceptible to activated astrocytes [17]. We observed an increased intensity of GFAP, demonstrating glial activation and neuroinflammation, closely surrounding spinal MNs in ALS patient tissues (Fig. 1j, k, *P* < 0.0001, Supplemental Fig. 1h–j). In addition, we demonstrated an increased presence of CD68+ cells, indicative of activated microglia, surrounding spinal MNs in ALS tissues (Supplemental Fig. 1k, *P* < 0.01). There was no increased GFAP immunoreactivity around OMNs (Fig. 1l, m; Supplemental Fig. 1l). Thus, OMNs persist in ALS, maintain overall normal MHC-I expression and are not exposed to the same inflammatory environment as spinal MNs. These results corroborate and extend upon previous findings revealing a reduction in HLA protein levels in spinal MNs in end-stage ALS patient tissues, which was shown to render MNs susceptible to activated astrocyte-toxicity [17]. Our data also support the hypothesis that HLA (MHC-1) downregulation is most strongly associated with neuronal populations undergoing degeneration. Our finding that the largest spinal MNs exhibit the lowest level of HLA protein and that this expression is lost in ALS, while the disease-resilient OMNs maintain HLA levels as a whole, constitute compelling correlations between selective vulnerability and resilience. Furthermore, our analysis demonstrating that the largest OMNs, which are maintained through disease, appear to lose HLA expression in ALS, indicates that loss of HLA alone is not sufficient to trigger MN degeneration.

### *β2m* and *HLA* mRNAs show compensatory upregulation in ALS which is not reflected in protein levels

To further explore the link between MHC-I signaling and differential MN susceptibility to ALS, we conducted a thorough investigation of *β2m* and *HLA* expression levels across MN subpopulations that show differential vulnerability to degeneration. We first reanalysed data from several published studies that isolated MNs from control tissues followed by microarray or RNA sequencing. This analysis showed that *β2m* and *HLA* mRNA expression levels were equal across disease-resistant OMNs and Onuf’s nucleus MNs and vulnerable spinal MNs in mouse ([20], *GSE52118*) (Supplemental Fig. 2a) and human ([24], GSE93939) (Supplemental Fig. 2b) ([19], *GSE40438*) (Supplemental Fig. 2c). Thus, the basal mRNA levels of *β2m* and *HLAs* in healthy neurons do not explain their differential susceptibilities to disease.

Neurons show both protective and detrimental responses to disease which can explain their early demise or resilience in ALS [21, 27, 32]*.* It has been reported that *β2m* mRNA levels increase with disease severity in spinal MNs of SOD1G93A mice [16]. This may seem contradictory to the loss of HLA protein in ALS ([17] and Fig. 1e)*,* but may reflect a compensatory upregulation of components of the MHC-I complex caused by the loss of the respective proteins. To shed further light upon this matter, we conducted bioinformatics analyses on published data sets comparing control and ALS tissues across MN subpopulations. We analysed our own RNA sequencing data sets [24, 25] and found that remaining spinal MNs in ALS tissues had a significantly higher level of *β2m* than MNs in control tissues (Fig. 1n, *P* = 0.03, *N* = 12 (Ctrl), *N* = 7(ALS)), while in OMNs levels remained unchanged and significantly lower than in ALS spinal MNs (Fig. 1n, *P* = 0.09, Welch t-test, *N* = 12 (Ctrl),* N* = 4 (ALS)). These findings highlight that maintaining low levels of *β2M* alone does not put MNs at risk. *HLA-A* and *HLA-C* levels were significantly higher in ALS spinal MNs than in control, and *HLA-A* was also induced in OMNs in ALS (Fig. 1n). Collectively, there appears to be a discordance between HLA protein and mRNA regulation in ALS patient tissues, with a compensatory upregulation of certain *HLA* mRNAs both in resilient and vulnerable MNs that is not reflected at the protein level.

**Fig. 2:**
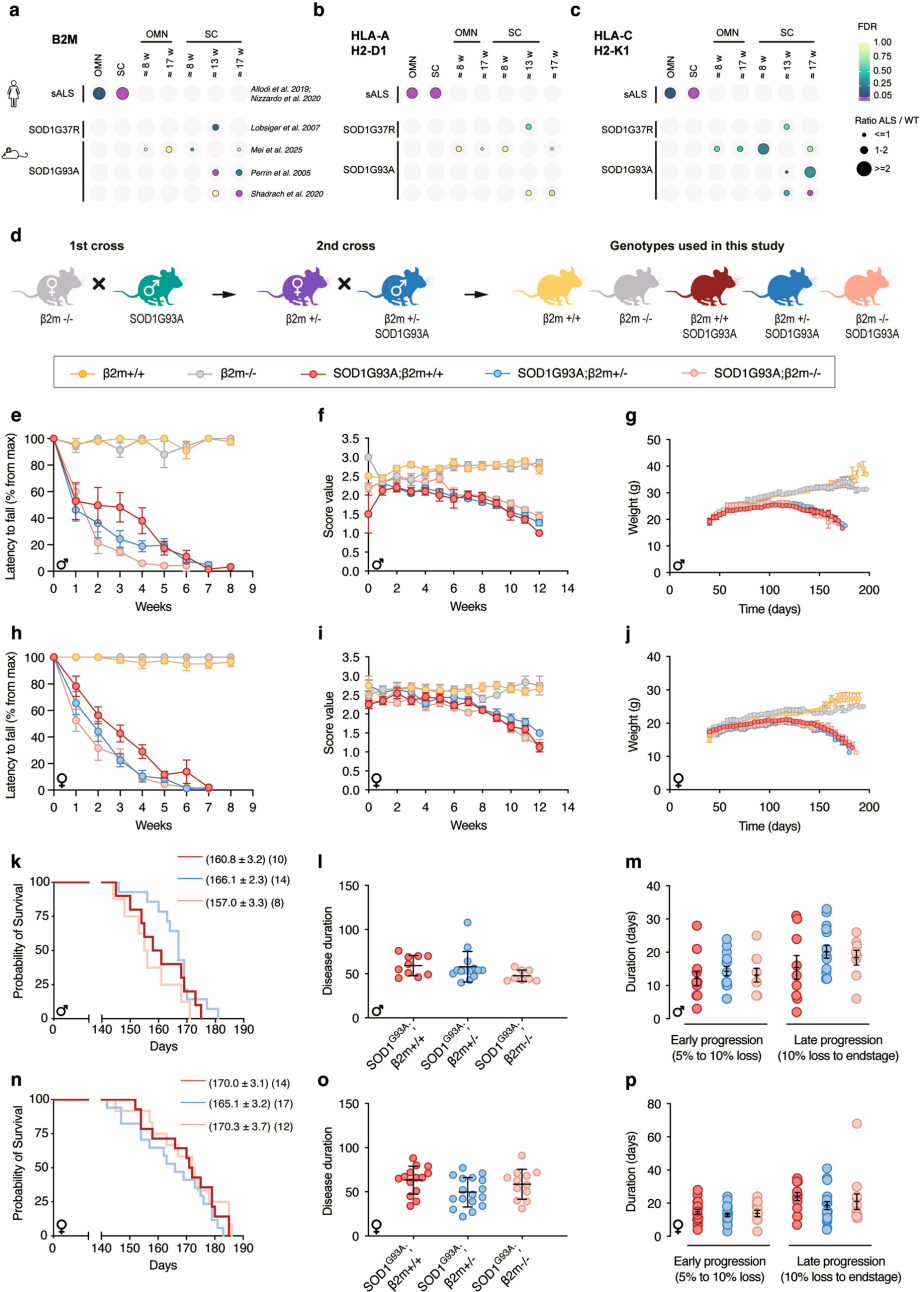
Loss of β2m does not impact survival or motor behaviour in ALS mice. **a**–**c** Summary of **a** *β2M*, **b** *HLA-A/H2-D1* and **c** *HLA-C/H2-K1* expression levels in OMN and spinal MNs (SC) across sporadic ALS (sALS) and mutant SOD1 ALS mice. **d** Schematic of mouse crosses to retrieve SOD1G93A mice lacking β2m. Motor performance was assessed by **e**, **h** the latency to fall in the grid test (N = 5–6 per group/sex) and **f**, **i** the extension reflex (N = 7–12 per group/sex) in male (**e**, **f**) and female (**h**, **i)** ALS mice with different doses of β2m. No difference was observed in males and females in the grid test and extension reflex with loss of β2m, neither was there an effect on weight loss in either sex (**g**–**j**). Analysis of survival showed no significant improvement in either sex with knockout of β2m in SOD1G93A mice (**k**, **n**). The disease duration was not affected in males (**l**) or females (**o**) when measured from the peak weight until end stage. Neither was there an effect when analysing early progression (5–10% weight loss from peak weight) and late progression (10% weight loss from peak weight until to end-stage) separately, in males (**m**) or females (**p**)

To better understand temporal regulation during disease progression, we next analysed transcriptome data sets from SOD1 ALS mouse models. Specifically, a microarray data set on lumbar MNs in SOD1G93A mice at an early symptomatic stage (P90;13 weeks) and a later symptomatic stage (P120;17 weeks) [21], showed an increase in *β2m* levels at the early stage (*P* = 0.01 Welch t-test, *N* = 3), but not at the later stage (*N* = 3) compared to wild-type littermates, likely due to the large variability in the control MNs at this age. Nonetheless, there was a significant increase of *β2m* mRNA in ALS MNs with disease progression (*P* = 0.04 Welch t-test, *N* = 3, Supplemental Fig. 2d). *H2-K1* levels remained low at P90 in ALS. Although not statistically significant at P120 when compared to wild-type (*P* = 0.16, Welch t-test, *N* = 3), an increase was observed between P90 to P120 in ALS (*P* = 0.007 Welch t-test, N = 3, Supplemental Fig. 2d), similar to the human equivalent, *HLA-C*, in end-stage sALS patient MNs (Fig. 1n). RNA sequencing data on spinal MNs from presymptomatic SOD1G85R mice at three months of age (show paralysis at 5–6 months) ([26]*, GSE38820*) revealed an apparent increase in *β2m* levels and *H2-D1* (*HLA-A*) with disease, while H2-K1 (*HLA-C*) remained unchanged (*N* = 2) (Supplemental Fig. 2e). A microarray data set on spinal MNs from 15 week, presymptomatic SOD1G37R mice (show paralysis at 25 weeks) [22] showed levels of *β2m*, *H2-D1* and *H2-K1* comparable to control (Supplemental Fig. 2f). In conclusion, our comprehensive transcriptome analysis showed a compensatory upregulation of *β2m* mRNA and *HLA-C* in spinal MNs in end-stage sALS tissues, and in symptomatic ALS mice (*H2-K1*, mouse equivalent to human *HLA-C*), while *HLA-A* was upregulated in both spinal MNs and OMNs in sALS patients (Fig. 1n), summarised in Fig. 2a–c.

Coupled with the intriguing correlation of HLA protein loss in the most vulnerable MNs, activation of astrocytes around these and the variable outcomes of β2m knockout in ALS mice published so far, made us pursue analysis of the effect of β2m loss in SOD1-ALS.

### Knockout of β2m has no impact on survival of SOD1G93A mice

To investigate the impact of β2m on ALS we crossed β2m knockout (β2m−/−) mice with SOD1G93A mice, both on congenic C57Bl/6 J backgrounds (Fig. 2d). We analysed motor performance by grid delay test and extension reflex. We did not observe any significant difference across the genotypes in the latency to fall (grid delay test) after maximum performance, in males (Fig. 2e, ANOVA (Kruskal–Wallis test followed by Dunn–Bonferroni post hoc correction), *N* = 7–12/group), or females (Fig. 2h, *N* = 7–12/group). Neither was there any difference in the extension reflex of either group or sex (Fig. 2f, i) (*N* = 7–12/group/sex). β2m−/− mice on a control background were comparable in performance to wild-type mice (Fig. 2e, f, h, i). All SOD1G93A mice showed a clear weight loss compared to non-transgenic littermates, as expected (Fig. 2g, j). There was no statistical difference in weights between the β2m genotypes on the SOD1G93A background, for males (Fig. 2g, *N* = 9–17/group) or females (Fig. 2j, *N* = 14–26/group). The loss of β2m did not give rise to any survival effect in male (Fig. 2k, *N* = 8–14/group) or female (Fig. 2n, *N* = 12–17/group) SOD1G93A mice (Mantel-Cox test). Analysis of disease duration based on peak weight measurements showed no difference in either sex, independent of if the disease progression was investigated in its entirety (Fig. 2l, o) or if analysis was done on early progression (5–10% weight loss) and late progression (10% weight loss to end-stage) separately (Fig. 2m, p).To investigate if end-stage ALS mice show a similar loss of MNs irrespective of the β2m genotype, and thus could be expected to have reached end-stage due to MN disease, spinal MNs were counted based on Nissl staining. Quantifications showed a clear decrease in MN numbers in SOD1G93A mice with no difference across β2m genotypes, compared to the β2m mice on a control background (which have a normal life-span) (Supplemental Fig. 3a–g, *N* = 4–5 mice (male and female) per group, *P* = 0.0006, one-way ANOVA).

Interestingly, β2m−/− mice on a wildtype background showed increased GFAP reactivity in their spinal cords compared to β2m+/+ littermates, at a level similar to SOD1G93A mice (Supplemental Fig. 3h, i, j, l). As these mice have a normal life-span, and retain all their MNs, it demonstrates that loss of HLA in combination with astrocyte activation is insufficient to cause an ALS phenotype. Loss of β2m caused an additional increase in GFAP reactivity in ALS mice (Supplemental Fig. 3j-l). Since SOD1G93A;β2m−/− lack infiltrating CD8+ T cells, which are seen in SOD1G93A mice (Supplemental Fig. 3j–l), we conclude that astrocyte activation appears uncoupled from immune infiltration, and that the additional GFAP reactivity does not exacerbate the disease further.

In conclusion, removing β2m from SOD1G93A mice and thus disabling MHC-I expression from all cells did not change the outcome of disease, indicating that β2m is not a major disease modifier in SOD1-ALS.

### Knockout of β2m preserves innervation of particular muscles in SOD1G93A ALS mice

**Fig. 3:**
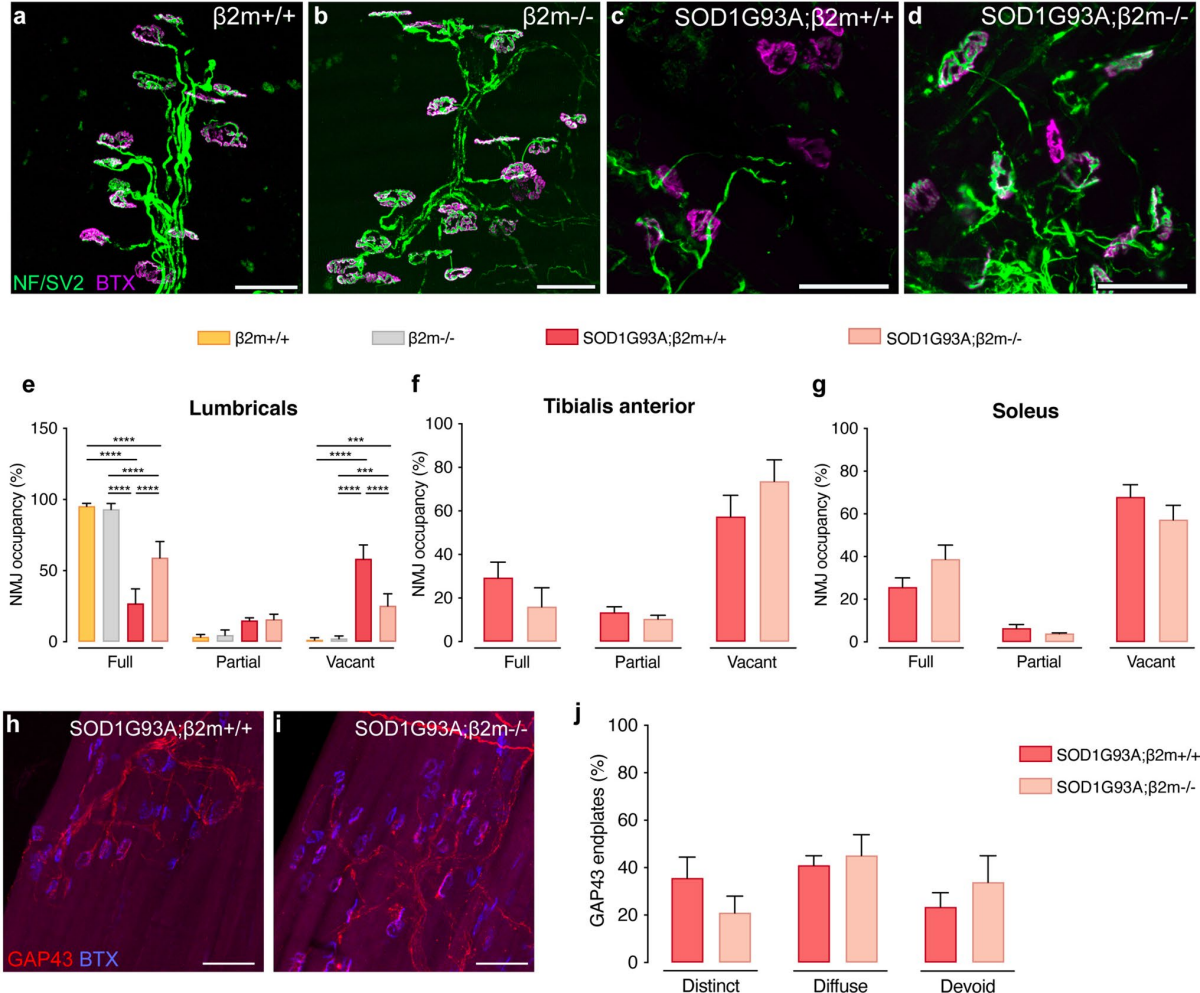
Knockout of β2m preserves innervation of particular muscles in SOD1G93A ALS mice. Representative images of the innervation pattern of motor nerves in lumbrical muscles of female **a** β2m+/+, **b** β2m−/−, **c** SOD1G93A;β2m+/+, or **d** SOD1G93A;β2m−/− mice at P140. The incoming motor nerves are visualized by a combined antibody staining against Neurofilament (2H3) and SV2A (green) and muscle endplates are stained with α-BTX (magenta). Quantification of innervation patterns, into fully occupied, partially occupied or vacant endplates was conducted in **e** lumbricals, **f** tibialis anterior and **g** soleus muscles. Lumbrical muscles in SOD1G93A;β2m−/− mice retained their innervation to a higher degree than in SOD1G93A;β2m+/+, as shown by **e** their higher level of full NMJ occupancy and lower percentage of vacant endplates (N = 4–5 female mice/group, *P* < 0.05, ANOVA; data are expressed as the mean ± SEM). Representative immunofluorescence images of lumbricals muscles from P140 female **h** SOD1G93A;β2m+/+ or **i** SOD1G93A;β2m−/− mice stained with the regeneration marker GAP43 (red). Muscle endplates were stained with α -BTX (blue). **j** Analysis of GAP43 innervation to muscle end plates showed no statistical significance between β2m genotypes in any of the innervation pattern classification (N = 4 mice/group. Statistical test: 2-way ANOVA; data are expressed as the mean ± SEM). Scale bars: 50 μm

To investigate if the removal of β2m in SOD1G93A had any impact on denervation, we conducted an analysis of NMJs in tibialis anterior, lumbricals and soleus muscles at P140. The incoming motor nerves were visualised using antibody staining against neurofilament (NEFM) and synaptic vesicle glycoprotein 2A (SV2A), while muscle endplates were labeled with α-BTX. Representative images of lumbrical muscles from female β2m+/+ (Fig. 3a), β2m−/− (Fig. 3b), SOD1G93A;β2m+/+ (Fig. 3c) and SOD1G93A;β2m−/− (Fig. 3d) mice at P140 illustrate these stainings. NMJ innervation patterns were defined as fully occupied, partially occupied or vacant endplates.

Analysis of lumbrical muscles demonstrated that innervation in β2m−/− mice was intact and comparable to β2m+/+ littermates. SOD1G93A;β2m−/− mice retained lumbrical innervation to a higher degree than SOD1G93A;β2m+/+ mice, as demonstrated by their higher level of fully occupied endplates and lower percentage of vacant endplates (Fig. 3a–e, *N* = 4–5 female mice/group, *P* < 0.0001, ANOVA). As all the mice on an ALS background performed similarly in the grid delay test, it is evident that this innervation did not result in a functional outcome in the test utilised. The retained innervation level of lumbricals appeared highly specific, as innervation in tibialis anterior (Fig. 3f) and soleus muscles (Fig. 3g) remained unchanged by the removal of β2m. To determine if the higher level of NMJ occupancy in lumbricals was due to preservation or rather sprouting and subsequent reinnervation of vacated endplates, we stained muscles with an antibody against GAP43 and endplates were again visualised by BTX. This analysis demonstrated that regeneration was similar in either genotype (Fig. 3h–j) and thus the higher level of occupied endplates in the SOD1G93A;β2m−/− is due to increased NMJ stability.

In conclusion, HLA and β2m are clearly regulated in vulnerable MNs in ALS, with loss of HLA protein and compensatory mRNA upregulation. Removal of β2m results in site-specific preservation of innervation, but is insufficient to impact motor behavior or survival in ALS mice.

## Discussion

The selective vulnerability of MNs is a hallmark of ALS, yet the underlying mechanisms remain unclear. While the large and fast-fatiguing MNs are selectively lost early in the disease, resistance is observed among MNs in the oculomotor and Onuf’s nuclei [33–35]. Building upon complex and even contradictory findings in existing literature, our study investigated the role of β2m and HLAs, which constitute the MHC-I complex, in dictating this selective vulnerability. We first validated the loss of the largest spinal MNs in end-stage ALS, and the persistence of OMNs. A key mechanistic insight from our study focuses on the differential basal expression of β2m and HLAs in MN subpopulations. By examining control donor tissue, we demonstrated an inverse correlation between HLA protein levels and MN size in the spinal cord, where the large, most vulnerable spinal MNs exhibited the lowest HLA expression. Beyond ALS, MHC-I dysregulation has been implicated in several neurodegenerative disorders. In Alzheimer’s disease, MHC-I destabilization by amyloid-β leads to synaptic dysfunction [12]. In Parkinson’s disease, upregulated neuronal MHC-I provokes autoimmune activation, rendering dopamine neurons more susceptible to neurodegeneration [36].

Staats et al. demonstrated that β2m expression was upregulated in MNs with disease progression in SOD1G93A ALS mice and that global deletion of β2m shortened survival. This led to the conclusion that β2m may play a protective role in ALS. The authors hypothesized that β2m promotes plasticity critical for muscle reinnervation, a compensatory mechanism that, when lost, accelerates muscle denervation and disease severity [16]. Song and colleagues on the other hand, demonstrated that MHC-I was downregulated on MNs in symptomatic SOD1G93A mice and on spinal MNs in end-stage ALS patient tissues [17]. As β2m levels were not analysed by *Song *et al. and HLA levels were not investigated by *Staats* and colleagues, these findings are not contradictory per se, but may just indicate that while HLA levels are reduced, there is compensation on the mRNA level by β2m to try to restore MHC-I levels.

Our broader transcriptome analysis across datasets indeed showed that both *β2m* and *HLA* mRNAs show compensatory upregulation in ALS, when HLA protein is gradually lost.

Reduced or low MHC-I levels may impair homeostatic or regenerative responses, aligning with its emerging non-immune functions in synaptic plasticity and axonal stability [5, 9] and render neurons more susceptible to inflammatory or excitotoxic insults.

In line with the findings of *Song* and colleagues, we show that HLA protein is markedly reduced in vulnerable spinal MNs in end-stage ALS patient tissues. We also demonstrate that OMNs retain HLA protein levels as a whole population, but that the largest OMNs, which are maintained throughout disease, lose HLA protein. This indicates that while loss of HLA protein in ALS is correlated with selective vulnerability, the loss of its expression alone is not sufficient to trigger MN degeneration.

Collectively, these findings strongly indicate that the selective vulnerability of spinal MNs in ALS is not dictated by differences in basal gene transcription of *β2m* or *HLA*, as we found that the mRNA levels of these genes were equal across vulnerable and resilient MNs in control donor tissue. Furthermore, in ALS donor tissue, our observation that spinal MNs upregulate β2m and HLA-C mRNAs, while HLA protein levels were decreased, suggests that post-transcriptional, translational, or localization mechanisms prohibit an increase of HLA protein levels, but this remains to be further investigated in detail. It also shows that vulnerable MNs develop an intrinsic, yet ultimately unsuccessful, protective response, which likely reflects a neuronal stress response, similar to what occurs after axonal injury [14, 15]. This aligns with comparative transcriptome data showing that vulnerable MNs in ALS react with both protective and degenerative gene programs. This was demonstrated by comparing the transcriptome data from *Mei* et al. [27] and *Shadrach* et al. [23], where the vulnerable MNs in ALS shared regeneration-associated factors, such as *Sox11, Gap43, Sprr1a, Adcyap, Chl1, Atf3,* and *Tubb3*, with MNs undergoing axonal repair after crush injury. This suggests that the upregulation of β2m and HLA transcripts could be part of a broader, but insufficient, intrinsic program initiated by the vulnerable MNs to promote plasticity and survival.

*Song* et al. demonstrated that ALS astrocytes reduced MHC-I expression on MNs, rendering these more vulnerable to astrocyte-mediated toxicity. Conversely, increasing MHC-I expression on MNs, for instance by overexpressing the human MHC-I molecule HLA-F, increased survival and improved motor performance in vivo. This established a paradigm where the maintenance of MHC-I expression is an active determinant of MN survival in the toxic ALS microenvironment. In line with these findings of an interplay between cell autonomous and non-cell autonomous mechanisms regulating vulnerability in ALS, we found no increase in astrocytic GFAP immunoreactivity around OMNs in ALS while the correlation between HLA protein levels and OMN size shifts from positive in control donors to inverse in ALS. This supports the idea that the preservation of a healthy glial milieu contributes to OMN resistance. These observations imply that in vulnerable spinal MNs, an early dysregulation of HLA expression may act as a trigger for pathological neuron-glia crosstalk, leading to glial activation and, ultimately, neurodegeneration. In contrast, the maintenance of overall HLA expression and the absence of reactive gliosis in OMNs likely protect these neurons from entering the same degenerative cascade. This aligns with the concept that non–cell-autonomous mechanisms, particularly astrocyte-induced toxicity, contribute to selective MN loss, and that their activation is induced by cell autonomous mechanisms in vulnerable neurons [37–40]. The differential loss of MHC-I protein in vulnerable and resistant MNs could be directly linked to these differences in local microenvironment, as *Song* et al. established that toxic ALS astrocytes reduce MHC-I expression on MNs, making these susceptible to astrocyte-induced cell death. Our finding that the largest, most vulnerable spinal MNs show the lowest HLA protein levels, combined with the neurotoxic environment created by activated spinal astrocytes, indicate that this puts them at a particular risk in ALS. It should however be pointed out that β2m knockout mice, which lack HLA in their MNs, showed an activated astrocyte response comparable to SOD1G93A ALS mice, without any subsequent MN loss. This indicates that β2m may guard astrocytes from becoming reactive in a controlled environment. Furthermore, from this result we can also conclude that loss of HLA in combination with astrocyte activation is not sufficient to induce MN degeneration. Here, further analysis comparing astrocytes and their secretome from β2m knockout mice and SOD1ALS mice may shed further light on differences in their toxicity towards MNs.

Finally, *Nardo* et al. [18] highlighted that MNs are particularly responsive in upregulating MHC-I molecules in response to insults, and this response is associated with preservation of axonal structure and function and slower disease progression in ALS mice. They demonstrated that MHC-I depletion accelerated disease onset and motor deficits but increased overall survival and promoted MN survival in SOD1G93A mice suggesting that MHC-I may play a deleterious role in disease survival. This depletion had different effects on the innervation depending on the targeted muscle groups.

Our current study introduces nuances to this paradigm, suggesting that the beneficial effects of β2m/MHC-I may be more localised than previously assumed, and provides insight into their role in selective vulnerability. Despite the role suggested by these molecular findings, functional genetic ablation of β2m in SOD1G93A mice did not improve motor performance, neither did it affect survival or MN loss. Nonetheless, some muscles, such as lumbrical muscles, did exhibit site-specific preservation of innervation, which underscores the muscle- and context-dependence of MHC-I. The preservation observed in fast-fatigable muscles like the lumbricals (typically among the first to degenerate) suggests that loss of β2m may indirectly stabilize NMJs in select circuits. This site-specific activity of MHC-I is reinforced by *Nardo* et al., where MHC-I depletion accelerated or delayed denervation depending on the targeted muscle groups. Taken together, these results support a model where MHC-I components, β2m and HLAs, are dynamically regulated in ALS, exerting a context-dependent role that varies across MN subtypes, disease stages, and peripheral targets.

While our data support a role for MHC-I in selective vulnerability, the functional impact of experimentally manipulating β2m in vivo has been strikingly inconsistent across studies. Four different studies, including ours [16–18] have evaluated the impact of removing β2m on disease progression in the SOD1G93A mouse model, resulting in distinct outcomes. Specifically, two studies reported a worsened phenotype and shortened survival [16, 17]; one reported an improved phenotype and increased survival [18]; and our study found no significant impact on behaviour, overall survival or MN loss. In those studies, both the SOD1G93A and the β2m knockout mice were reported to be on a C57BL/6 background. The striking message from this comparison is that the C57BL/6 substrains and/or non-genetic variables appear to have a greater impact on the disease phenotype. It is well-documented that the longevity of mouse strains, even within the C57BL/6 lineage, can lead to phenotypic differences. As highlighted by The Jackson Laboratory (JAX), the longer time that strains are separated from each other, the greater the number of genetic differences between them, which can lead to large phenotypic differences (JAX, 2016, [41]. In their seminal work, *Nardo* et al. [42], demonstrated that SOD1G93A mice on distinct genetic backgrounds (C57BL/6 vs. 129 Sv) show consistent differences in disease progression and lifespan. Crucially, the slower-progressing C57BL/6 strain exhibited a striking upregulation of immune system processes and increased MHC-I expression in MNs at disease onset, contrasting with the rapidly progressing 129 Sv strain. This establishes that the genetic background modulates key pathways which may then override the subtle experimental effect of the β2m knockout in the different studies. Furthermore, differences in microbiota, environmental stressors, or facility-specific pathogens could further modulate the immune-neuronal interplay and thereby the β2m phenotype. Those could influence the outcome, as the animal’s immune status is impacted by the loss of β2m, a key variable not investigated in any of the studies. Lastly, the high degree of genetic drift among B6 substrains is known to influence neuroinflammatory and metabolic traits (JAX Blog, 2016), reinforcing the conclusion that the background of the animal and immune status likely mattered more than the loss of β2m itself.

Taken together, our findings provide new insights into the complex, context-dependent regulation of MHC-I in ALS. We demonstrate that the HLA protein levels are determinant of MN vulnerability, with the most susceptible MNs exhibiting the lowest HLA protein levels. The observed β2m and *HLA-C* mRNA upregulation in vulnerable spinal MNs likely represents a compensatory mechanism that, despite the concurrent activation of regenerative factors [27], is ultimately overcome by the disease. Our functional in vivo study on SOD1G93A ALS mice lacking β2m suggests that β2m removal does not have a profound, singular effect on disease outcome or functional motor performance, while histological analysis suggests a modest benefit on NMJ stability with partial preservation of innervation in particular muscles. This indicates that the intrinsic protective mechanisms mediated by β2m are insufficient to reverse the fatal trajectory of ALS. It is plausible that while MHC-I can protect the distal axon, a function aligned with its role in synaptic plasticity and nerve regeneration [18], this localized protection is not sufficient to counteract the chronic, progressive, and non-cell autonomous toxicity exerted by surrounding glia and mutant proteins [17]. Critically, our comparison with existing literature underscores the overriding influence of genetic and environmental context on ALS phenotypes in mouse models, as demonstrated by the variable effects of β2m removal, likely depending on genetic background and environment.

## Supplementary Information

Below is the link to the electronic supplementary material.

Supplementary file1 (PDF 4882 KB)

## Data Availability

Published microarray datasets processed within this manuscript are available from GEO at https://www.ncbi.nlm.nih.gov/query; (GSE40438, [19]*;* GSE52118, [20], GSE162028, [23]) and processed data sets from [21, 22, 24, 25] were retrieved from the authors. Raw datasets from [26, 27] are available at GEO https://www.ncbi.nlm.nih.gov/query; *GSE38820* and GSE244538.
