# Supplementary material for: Reevaluating the role of beta2-microglobulin: new insights on selective vulnerability in ALS pathology
Source: Acta Neuropathol. 2026 May 29;151(1):63. doi: 10.1007/s00401-026-03024-3 (PMC13221346; doi:10.1007/s00401-026-03024-3)
Supplement: Supplementary file 1 — Supplementary file1 (PDF 4882 KB) [file 401_2026_3024_MOESM1_ESM.pdf]

Supplementary material

Reevaluating the Role of Beta2-Microglobulin: New Insights on Selective Vulnerability in ALS Pathology

Leboeuf Melanie[1,2], Nijssen Jik[2,3], Comley Laura Helen[3], Aguila Benitez Julio Cesar[3], Mei Irene[1], Gómez Alcalde Silvia[1], Muñoz de Bustillo-Alfaro Ramón A.[4], Radoi Vlad[1], Nichterwitz Susanne[1], Schweingruber Christoph[1,2], Acevedo Arozena Abraham[4], Hedlund Eva[1,2,3, †] and Cullheim Staffan[3, †]

[†]These authors contributed equally to this work.

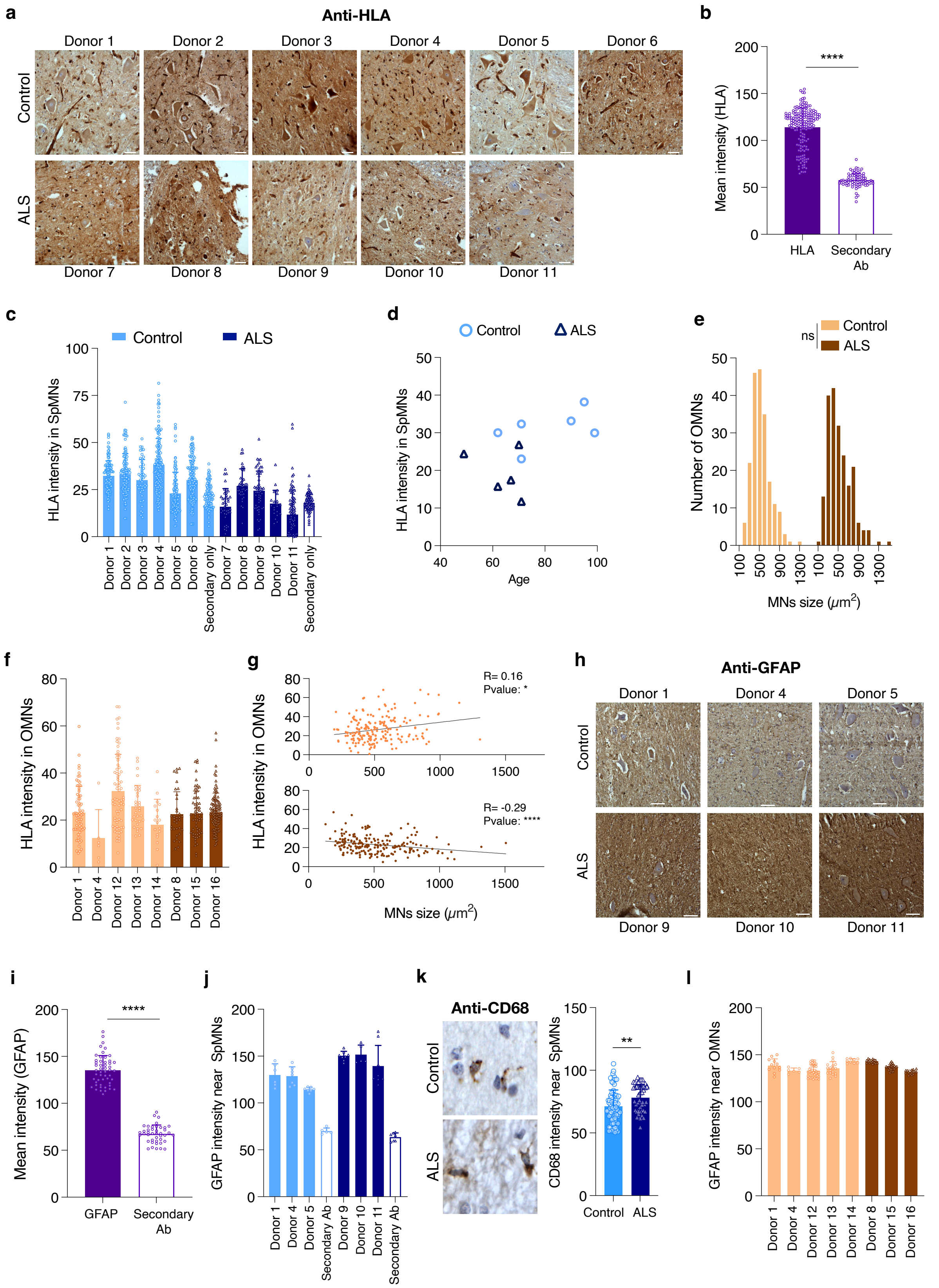

Supplemental Figure 1. HLA protein level is decreased in MNs in ALS patient tissues while GFAP intensity surrounding MNs is increased. (a) Immunohistochemical staining

against HLA-ABC in human *post mortem* spinal cords from control and ALS donors. (**b**) Negative controls (secondary antibody only, without primary antibody) demonstrated substantially decreased staining intensity compared to the sections stained with HLA-ABC antibody (**c**) HLA MN intensity across spinal MNs (SpMNs) in individual donor tissues shows a decrease in HLA protein levels in ALS compared to control. (**d**) HLA SpMN intensity shows no correlation with tissue donor age. The spearman correlation between increasing age and HLA levels in spinal MNs were *R=0.2, P*=0.78 for control tissues and *R=0.3, P*=0.68 for ALS tissues. (**e**) There was no significant loss of OMNs in ALS patient tissues or change in soma sizes. (P=0.9993, Kolmogorov-Smirnov test) (**f**) Quantification of HLA protein expression across OMNs in individual donors remained unchanged with ALS. (**g**) HLA immunoreactivity and OMN size are positively correlated in control *post mortem* donor tissues but inversely correlated in ALS tissues. (Ctrl: R=0.16, P=0.0210, Spearman correlation, ALS: R=-0.29, P<0.0001, Spearman correlation). (**h)** Staining against GFAP to visualize reactive astrocytes in three control and three ALS donor spinal cord *post mortem* tissues. (**i**) Negative controls (secondary antibody only, without primary antibody) demonstrated substantially decreased staining intensity compared to the sections stained with GFAP antibody. (**j**) Quantification of GFAP intensity across SpMNs in individual control and ALS donor spinal cord tissues shows an increase in GFAP levels with ALS (**k**) Staining and quantification of CD68 intensity around SpMNs in control and ALS donor shows an increase in CD68 levels with ALS confirming the innate immune reaction. (**l**) There was no increase in GFAP intensity around OMNs in end-stage ALS patient tissues. Data are expressed as the mean ± SD.

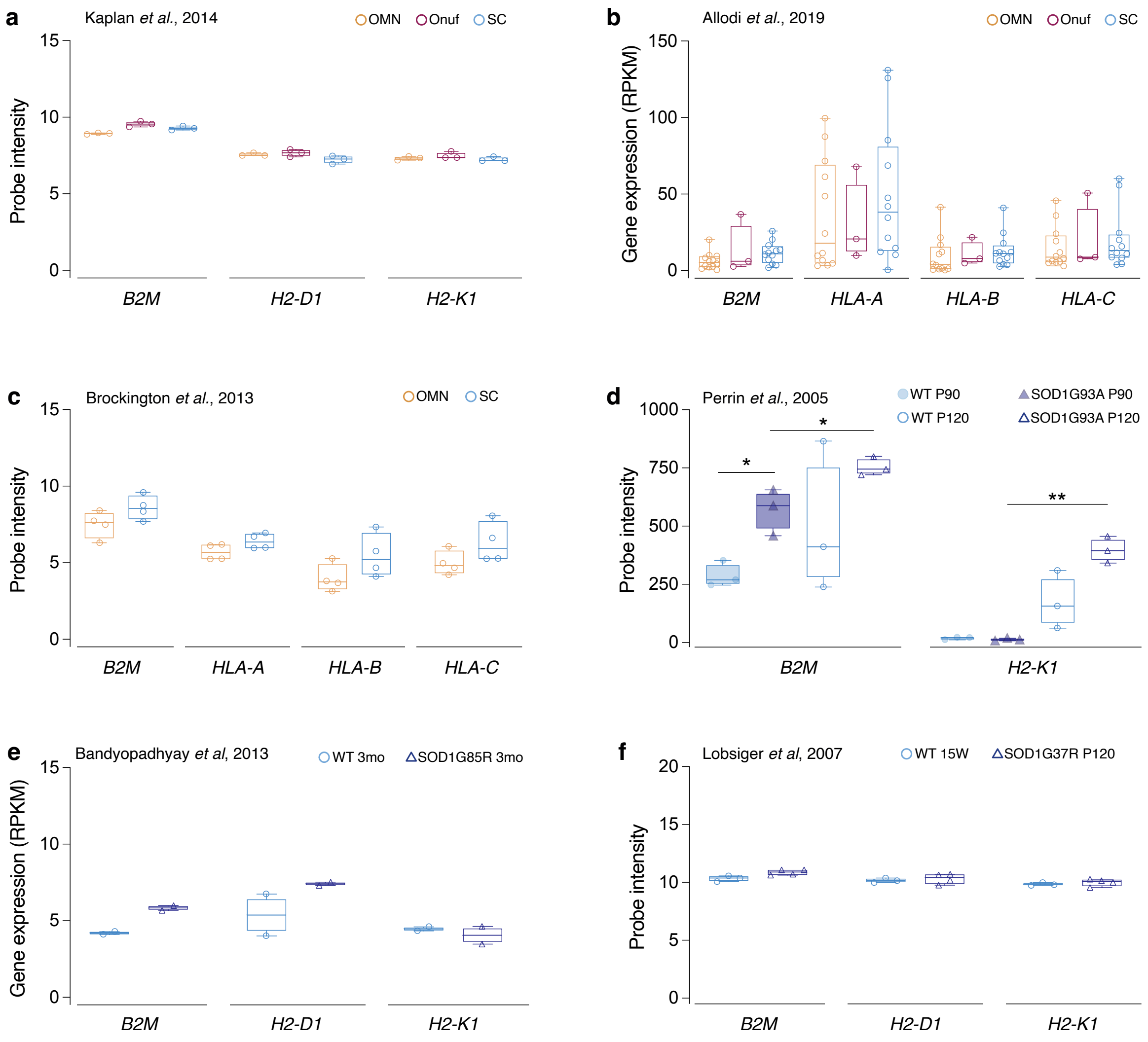

Supplemental Figure 2. β2m and HLA levels do not underlie differential neuronal vulnerability across brain stem and spinal cord motor nuclei, but are regulated in vulnerable neurons in response to increased disease burden. Analysis of *β2m*, and *HLA* mRNA levels across control data sets show no statistically significant difference across motor neuron subpopulation with differential vulnerabilities to degeneration in ALS in (**a**) mouse (Kaplan et al. 2014) or (**b, c**) human (Allodi et al. 2019; Brockington et al. 2013). Overexpression of mutant SOD1 in mice causes differential upregulation of β2m and HLA, which may depend on disease stage and mutation, as shown in (**d**) SOD1G93A mice at presymptomatic (P90) and symptomatic (P120) stages (Perrin et al. 2005) and (**e**) SOD1G85R mice at 3 months (Bandyopadhyay et al. 2013) and (**f**) presymptomatic SOD1G37R mice at 15 weeks (Lobsiger et al. 2007). Data are expressed as the median ± min and max. Whiskers extend to 1.5x the interquartile range (IQR).

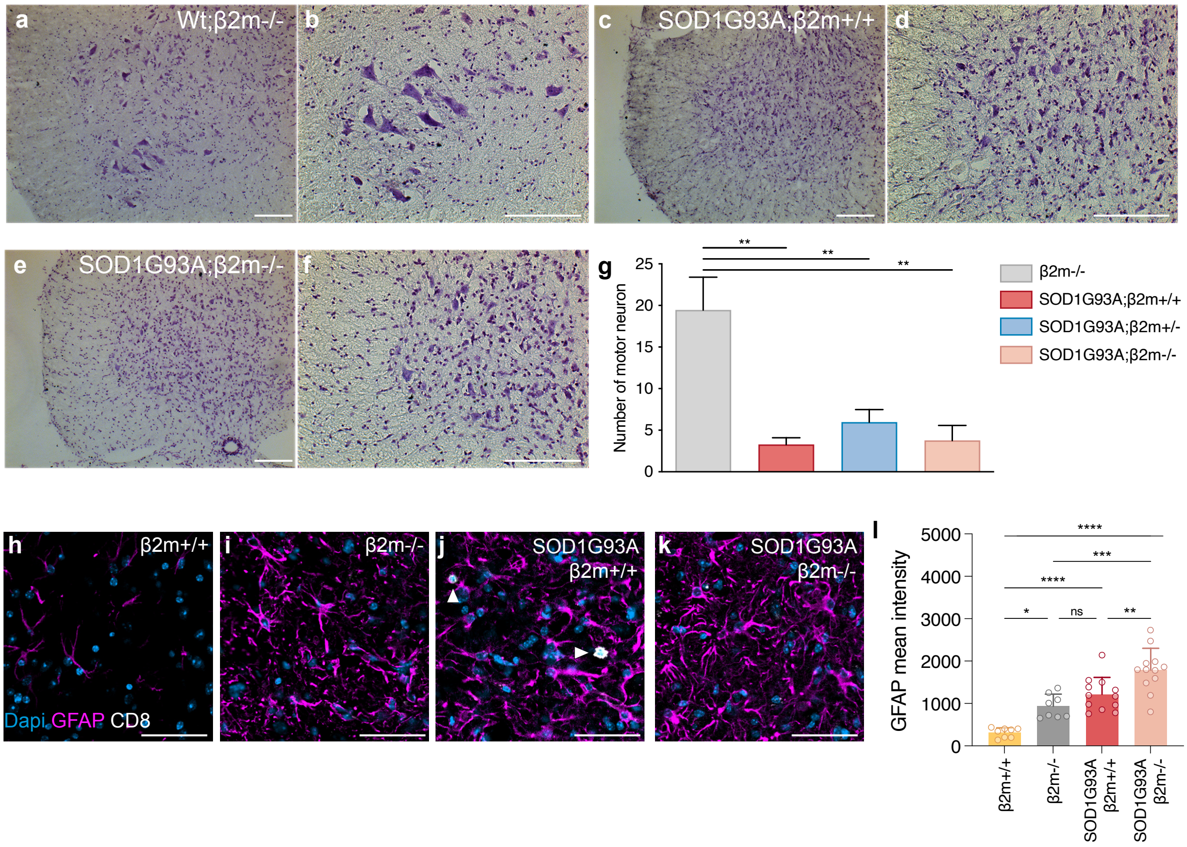

Supplemental Figure 3. Loss of β2m does not impact the level of motor neuron loss in SOD1G93A ALS mice. Quantification of spinal motor neurons based on Nissl staining of spinal cord sections across genotypes, with representative bright field images from (**a, b**) β2m-/- mice, (**c, d**) SOD1G93A;β2m+/+ mice and (**e, f**) SOD1G93A;β2m-/- mice. (**g**) Summary graph of motor neuron counts showing significance between β2m-/- mice on a wild-type (C57Bl/6) background (*P=0.0006*, one-way ANOVA; data are expressed as the mean ± SEM) and SOD1G93A ALS mice, while no difference was observed between the β2m genotypes in the SOD1G93A ALS mice. Quantifications were done at end-stage for SOD1G93A mice across β2m genotypes and at a matched time point for β2m mice on a control background, which have normal life-span. N=4-5 mice per group (combined male and females). Stainings against GFAP and CD8 across genotypes, with representative images from (**h**) β2m+/+ mice, (**i**) β2m-/- mice, (**j**) SOD1G93A;β2m+/+ mice and (**k**) SOD1G93A;β2m-/- mice. Scale bars: 50um. (**l**) Quantification of GFAP levels around SpMNs in the different mice genotypes.

Supplemental Table S1. The number of human post mortem tissue samples used and MNs quantified for anti-HLA intensity (relating to Supplementary Figure S1).

	Spinal MNs		OMNs	
	Control	ALS	Control	ALS
Number of donors	6	5	5	3
Number of sections	24	15	11	6
Number of cells	550	198	198	158

Supplemental Table S2. The number of human post mortem tissue samples and sections as well as images captured (per staining) that were used to quantify anti-GFAP and anti-CD68 intensity (relating to Supplementary Figure S1).

	Spinal MNs		OMNs	
	Control	ALS	Control	ALS
Number of donors	3	3	5	3
Number of sections	15	12	11	6
Number of images	20	21	28	19

References

Allodi, Ilary, Jik Nijssen, Julio Aguila Benitez, et al. 2019. « Modeling Motor Neuron Resilience in ALS Using Stem Cells ». *Stem Cell Reports* 12 (6): 1329-41. https://doi.org/10.1016/j.stemcr.2019.04.009.

Bandyopadhyay, Urmi, Justin Cotney, Maria Nagy, et al. 2013. « RNA-Seq Profiling of Spinal Cord Motor Neurons from a Presymptomatic SOD1 ALS Mouse ». *PLOS ONE* 8 (1): e53575. https://doi.org/10.1371/journal.pone.0053575.

Brockington, Alice, Ke Ning, Paul R. Heath, et al. 2013. « Unravelling the Enigma of Selective Vulnerability in Neurodegeneration: Motor Neurons Resistant to Degeneration in ALS Show Distinct Gene Expression Characteristics and Decreased Susceptibility to Excitotoxicity ». *Acta Neuropathologica* 125 (1): 95-109. https://doi.org/10.1007/s00401-012-1058-5.

Kaplan, Artem, Krista J. Spiller, Christopher Towne, et al. 2014. « Neuronal Matrix Metalloproteinase-9 Is a Determinant of Selective Neurodegeneration ». *Neuron* 81 (2): 333-48. https://doi.org/10.1016/j.neuron.2013.12.009.

Lobsiger, Christian S., Séverine Boillée, et Don W. Cleveland. 2007. « Toxicity from different SOD1 mutants dysregulates the complement system and the neuronal regenerative response in ALS motor neurons ». *Proceedings of the National Academy of Sciences* 104 (18): 7319-26. https://doi.org/10.1073/pnas.0702230104.

Perrin, Florence E., Gaelle Boisset, Mylene Docquier, Olivier Schaad, Patrick Descombes, et Ann C. Kato. 2005. « No Widespread Induction of Cell Death Genes Occurs in Pure Motoneurons in an Amyotrophic Lateral Sclerosis Mouse Model ». *Human Molecular Genetics* 14 (21): 3309-20. https://doi.org/10.1093/hmg/ddi357.
